# Diagnostic value of LncRNA JPX in osteoporotic fracture (OPF) and its role in inhibiting osteogenic differentiation by targeting miR-219a-5p

**DOI:** 10.1186/s41065-026-00685-8

**Published:** 2026-05-01

**Authors:** Junli Li, Yiwei Li, Ningning Zhang, Zhijian Cheng

**Affiliations:** 1 Endocrinology and Metabolic Diseases Department, Yantai Yantaishan Hospital, Yantai, 264003 China; 2 Department of Orthopedics, Pan’an People’s Hospital, Pan’an, 322300 China; 3 Department of Orthopedics, Affiliated Hospital of Hebei University of Engineering, Handan, 056000 China; 4 Department of Orthopedics, The Second Affiliated Hospital of Xi’an Jiaotong University, No. 157, West Fifth Road, Xi’an, 710004 China

**Keywords:** JPX, miR-219a-5p, Osteoporotic fractures, Fracture healing

## Abstract

**Background:**

This study was designed to investigate the diagnostic potential of the long non-coding RNA JPX in osteoporotic fractures (OPF) and to explore its functional role in the regulation of fracture healing.

**Methods:**

A total of 244 patients with osteoporosis were enrolled, including 110 with osteoporosis (OP) and 134 with OPF. Expression levels of JPX, miR-219a-5p, and osteogenic markers (OPG, ALP, Collagen I, OCN) were detected by RT-qPCR. ROC analysis was employed to assess the clinical value of JPX. In human bone marrow mesenchymal stem cells (hBMSCs), gain- and loss-of-function experiments were performed to modulate JPX and miR-219a-5p expression. Cell proliferation was evaluated by CCK-8 assay. Flow cytometry assessed apoptosis. Dual luciferase reporter assays and RIP experiments validated the targeting relationship between JPX and miR-219a-5p.

**Results:**

JPX was upregulated in the OPF group, with good diagnostic performance (AUC = 0.897; specificity = 76.87%; sensitivity = 84.55%), and JPX was identified an independent predictive factor. DFH and NFH could also be distinguished. JPX upregulation inhibited proliferation, promoted apoptosis, and suppressed osteogenic markers and ALP activity. JPX largely resides in the cytoplasm and directly binds miR-219a-5p, forming a ceRNA axis; upregulation of miR-219a-5p could reverse these effects.

**Conclusion:**

JPX is associated with fracture risk and delayed healing, potentially influencing osteogenesis by sponge-like regulation of miR-219a-5p.

**Supplementary Information:**

The online version contains supplementary material available at 10.1186/s41065-026-00685-8.

## Introduction

Osteoporosis (OP) is one of the most common and challenging orthopedic diseases in the context of global aging [1, 2]. Its core features include reduced bone mass, damage to bone microarchitecture, and increased bone fragility, all of which markedly raise the risk of osteoporotic fractures (OPF) [3]. Studies show a significant gender difference in the lifelong remaining risk of OPF for individuals around age 50: about 50% for women and 20% for men [4]. OPF not only substantially increases mortality and disability but also imposes a heavy burden on social medical resources [5]. Although current diagnostic and therapeutic approaches for OPF are rapidly expanding, fracture healing remains frequently constrained by inflammatory environments and diminished regenerative capacity after injury, among other factors [6]. This is especially true in patients with delayed union or nonunion, where the healing process is complex and carries high risks [7]. Targeting this challenge, exploring new molecular mechanisms and potential targets to promote osteogenic repair holds important theoretical value and clinical significance.

In recent years, the regulatory roles of long noncoding RNAs (lncRNAs) in bone metabolism and fracture repair have been gradually revealed [8]. For example, lncRNA SNHG14 modulates the balance between osteogenic and adipogenic differentiation of bone marrow mesenchymal stem cells (BMSCs) via the miR-27a-3p/LMNB1 axis, influencing the progression of osteoporosis [9, 10]. lncRNA NEAT1 is a key mediator of BMSC differentiation, and it disrupts mitochondrial function and stem cell pluripotency, leading to imbalanced cell fate decisions [11]. JPX, as a critical lncRNA, shows regulatory roles in cell proliferation, apoptosis, and signaling pathway activity across various tumors and bone-related diseases [12]. At the level of skeletal muscle, JPX expression is upregulated in BMSCs under microgravity, suggesting its potential involvement in disuse osteoporosis mechanistic pathways [13]; in osteosarcoma cell lines, nucleus pulposus cells, and hematologic disease samples, JPX influences cell vitality, metastasis, and differentiation through distinct miRNA axes [14, 15]. These findings suggest that JPX may regulate osteoblast proliferation, differentiation, and apoptosis via interactions with miRNA networks, thereby affecting the healing process of OPF.

miR-219a-5p is an important miRNA in bone metabolism regulation, and recent studies show its pivotal role in osteogenic differentiation. In age-related bone samples, miR-219a-5p levels decline, and overexpression can enhance osteogenic activity [16]; in osteosarcoma and osteoarthritis, miR-219a-5p modulates the function of relevant cellular models by targeting key signaling pathways [17, 18]. Nevertheless, the precise role of miR-219a-5p in OPF and fracture healing remains incompletely understood, especially whether JPX modulates osteogenic differentiation directly or indirectly via miR-219a-5p, which requires systematic elucidation.

Based on the available evidence, we propose that JPX may serve as a potential biomarker for OPF, and that the “JPX/miR-219a-5p axis” may participate in the molecular mechanisms governing osteoblast growth and differentiation. This hypothesis was validated through clinical data analysis and in vitro cellular experiments, aiming to provide novel diagnostic markers and therapeutic targets for OPF and fracture healing.

## Materials and methods

### Patient enrollment

The study enrolled a total of 244 patients diagnosed with osteoporosis at the Affiliated Hospital of Hebei University of Engineering from March 2020 to October 2023. Among them, 110 had OP alone and 134 had OPF. Osteoporosis was diagnosed according to the World Health Organization (WHO) bone mineral density (BMD) criteria, with a T-score ≤ -2.5. Exclusion criteria included: [1] pathological or old fractures; [2] malignancy; [3] severe hepatic or renal impairment; [4] long-term use of medications known to affect bone metabolism; [5] immune deficiencies or coagulation disorders. All participants were informed about the nature and potential consequences of the study and provided written informed consent. Each participant provided 5 mL of peripheral blood for serum separation. Follow-up lasted 4 months. At 4 months, patients were re-evaluated; if X-ray examination showed the fracture ends with only minor callus formation, mild local demineralization, a clearly visible fracture line, and no obvious bone sclerosis, they were classified into the delayed healing fracture (DFH) group. Participants were then observed for an additional 2 weeks; if no deformity occurred at the fracture site, they were categorized into the normal fracture healing (NFH) group. The final grouping was: 98 normal healing cases and 36 delayed healing cases. The study received approval from the Affiliated Hospital of Hebei University of Engineering ethics committee and was conducted in accordance with the principles of the Declaration of Helsinki.

### Cell culture and transfection

Human bone marrow-derived mesenchymal stem cells (hBMSCs) were purchased from BeNa Culture Collection (BNCC, China) and cultured in Mesenchymal Stem Cell Medium (MSCM) supplemented with 10% fetal bovine serum (FBS) and 1% penicillin/streptomycin. Cells were maintained at 37 °C in a humidified atmosphere containing 5% CO₂. Osteogenic differentiation was induced using osteogenic medium containing 50 µg/mL ascorbic acid (Sigma-Aldrich, USA), 5 mM β-glycerophosphate (Sigma-Aldrich, USA), and 10 nM dexamethasone (Sigma-Aldrich, USA).

Seed hBMSCs into 6-well plates at a density of 2 × 10⁵ cells per well. Once cell confluence reached 75–85%, perform transfection. Targeted JPX-specific siRNA and negative control (si-NC), as well as JPX overexpression plasmids (oe-JPX) and their controls (oe-NC), were obtained from GenePharma (China). hBMSCs were detached with trypsin and transfected with Lipofectamine 2000 (Invitrogen, USA) along with miR-219a-5p mimics and mimic NC (RiboBio, China), either alone or co-transfected with the plasmids. After 48 h, downstream analyses were performed.

### RNA extraction and reverse transcription-quantitative PCR (RT-qPCR)

Total RNA was extracted from serum samples and hBMSCs using TRIzol reagent (Invitrogen, USA). RNA was then reverse-transcribed into cDNA using a reverse transcription kit (Invitrogen, USA). Expression levels of JPX, miR-219a-5p, osteoprotegerin (OPG), alkaline phosphatase (ALP), Collagen I, and osteocalcin (OCN) were measured by real-time quantitative PCR using SYBR Premix Ex Taq II (Takara, Japan) on a real-time PCR system. GAPDH and U6 were used as internal reference genes, and the 2 − ΔΔCt method was employed for normalization.

### Dual-luciferase reporter assay

The ENCORI database predicted binding sites between JPX and miR-219a-5p. A dual-luciferase reporter assay (KeyGEN Biotech, China) was used to analyze the interaction between JPX and miR-219a-5p. Putative miR-219a-5p binding sites in JPX were cloned into the pGL3 vector to create wild-type (WT) and mutant (MUT) reporter constructs. These recombinant vectors were transfected into hBMSCs, and in some groups, miR-219a-5p mimics were co-transfected. After 48 h, firefly luciferase activity was measured using a luminometer (Thermo Fisher Scientific, USA), and Renilla luciferase activity was used for normalization.

### RNA pull-down assay

Biotin-labeled Bio-NC or Bio-miR-219a-5p (RiboBio, China) were transfected into hBMSCs at 50 nM. After 48 h, cells were harvested and lysed. The lysates were incubated at 4 °C with M-280 magnetic Dynabeads (Invitrogen, USA) pre-blocked with RNase-free BSA and yeast tRNA for 3 h. The beads were washed sequentially with cold lysis buffer, low-salt buffer (three times), and high-salt buffer (once). RNA bound to the beads was extracted with Trizol, and JPX expression was quantified by RT-qPCR.

### Cell counting kit-8 (CCK-8) assay

Cell proliferation was assessed with a CCK-8 kit (Dojindo Laboratories, Japan). Briefly, transfected hBMSCs were seeded at 5 × 10³ cells per well in 96-well plates. At 0, 24, 48, and 72 h, 10 µL of CCK-8 solution was added per well, followed by 2 h of incubation. Absorbance was measured at 450 nm using a microplate reader (Thermo Fisher Scientific, USA).

### Flow cytometry

Cells from all groups were washed with PBS and stained with Annexin V-PE/7-AAD apoptosis staining kit (BD Biosciences, USA). Apoptosis was analyzed by flow cytometry (FACScan, BD Biosciences, USA), distinguishing early and late apoptotic cells. Data were processed with BD CSampler software.

### ALP activity assay

Seed the cells into a 24-well plate at a density of 5 × 10⁴ cells per well. ALP activity was measured using an ALP assay kit (Beyotime, China) with an enzyme-linked immunoassay reader (BioTek, USA) at 405 nm to determine optical density (OD) values.

### Nuclear and cytoplasmic fractionation

Nuclear and cytoplasmic RNAs were separated using the SurePrep Nuclear or Cytoplasmic RNA Purification Kit (Fisher Scientific, USA). Briefly, hBMSCs were lysed with a lysis buffer to disrupt the cell membrane, and RNA was extracted from the nucleus and cytoplasm using a cell disruption and fractionation apparatus. JPX expression was then measured by RT-qPCR in each fraction.

### Western blot

For cell samples, total protein was extracted using RIPA lysis buffer (R0010, Solarbio, Beijing, China) containing a mixture of protease inhibitors. Equal volumes of protein samples were separated by electrophoresis on SDS-PAGE gels of appropriate concentrations (10% or 15%). Subsequently, proteins were transferred to a PVDF membrane (0.22 μm, Millipore) using a wet transfer system (Bio-Rad). After transfer, the membrane was blocked with blocking buffer at room temperature for 30 min. The membrane was then incubated with the corresponding primary antibody at 4 °C overnight. The following day, the membrane was incubated with the secondary antibody at room temperature for 1 h. Finally, protein bands were detected using ultra-sensitive ECL chemiluminescent substrates. Relative quantification of band intensities was performed using ImageJ software (version 1.52a).

The primary antibody information is as follows: GAPDH (#380646, ZenBio, China; 1:7500), ALP (#ab229126, Abcam; 1:1000), OPG (#bs-20624R, Bioss; 1:1000), OCN (#ab133612, Abcam; 1:1000), Collagen I (#ab260043, Abcam; 1:1000). Uncropped blots are provided in *Supplementary materials*.

### Statistical analysis

All data were analyzed using GraphPad Prism 9 or SPSS 22. Results are presented as mean ± standard deviation. Between-group differences were assessed by Student’s t-test or one-way ANOVA, with post hoc comparisons performed using Dunnett’s test or Tukey’s test. A p-value < 0.05 was considered statistically significant.

## Results

### Baseline characteristics of participants

The study first analyzed the clinical data of participants. There were statistically significant differences between the OP group and the OPF group in T-score and 25-(OH) vitamin D (*P* < 0.05). There were no significant differences in age, sex, BMI, smoking status, alcohol use, or family history of osteoporosis (*P* > 0.05; Table 1). The distributions of these clinical characteristics between the NFH and DFH groups were consistent with those observed when comparing the OP and OPF groups (Table 2).

**Table 1:** Comparison of clinical data between the OP and OPF patients

Items	OP (*n* = 110)	OPF (*n* = 134)	*P*
Age, years	61.28 ± 10.46	62.98 ± 10.63	0.213
BMI, kg/m^2^	23.41 ± 3.05	24.08 ± 3.52	0.125
Gender (male/female)	31/79	34/100	0.623
Smoking (no/yes)	66/44	77/57	0.690
Drinking (no/yes)	48/62	68/66	0.270
Family history of osteoporosis(no/yes)	111	106	0.652
Bone density (T score)	-2.80 ± 0.72	-3.29 ± 0.97	< 0.001
25-(OH) vitamin D (ng/mL)	26.08 ± 4.26	18.10 ± 3.01	< 0.001

*OP* Osteoporosis, *OP*F Osteoporotic fracture, *BMI*  Body Mass Index

**Table 2:** Comparison of clinical data between the NFH and DFH patients

Items	NFH (*n* = 98)	DFH (*n* = 36)	*P*
Age, years	63.23 ± 10.61	62.27 ± 10.82	0.646
BMI, kg/m^2^	24.04 ± 3.64	24.17 ± 3.23	0.858
Gender (male/female)	24/74	10/26	0.701
Smoking (no/yes)	50/40	19/17	0.510
Drinking (no/yes)	48/50	20/16	0.503
Family history of osteoporosis(no/yes)	39/59	12/24	0.498
Bone density (T score)	-3.16 ± 1.02	-3.64 ± 0.73	0.011
25-(OH) vitamin D (ng/mL)	18.44 ± 2.95	17.20 ± 3.01	0.035

*NFH* normal fracture healing, *DFH* delayed fracture healing, *BMI* Body Mass Index

### Expression of JPX and diagnostic value

**Fig. 1:**
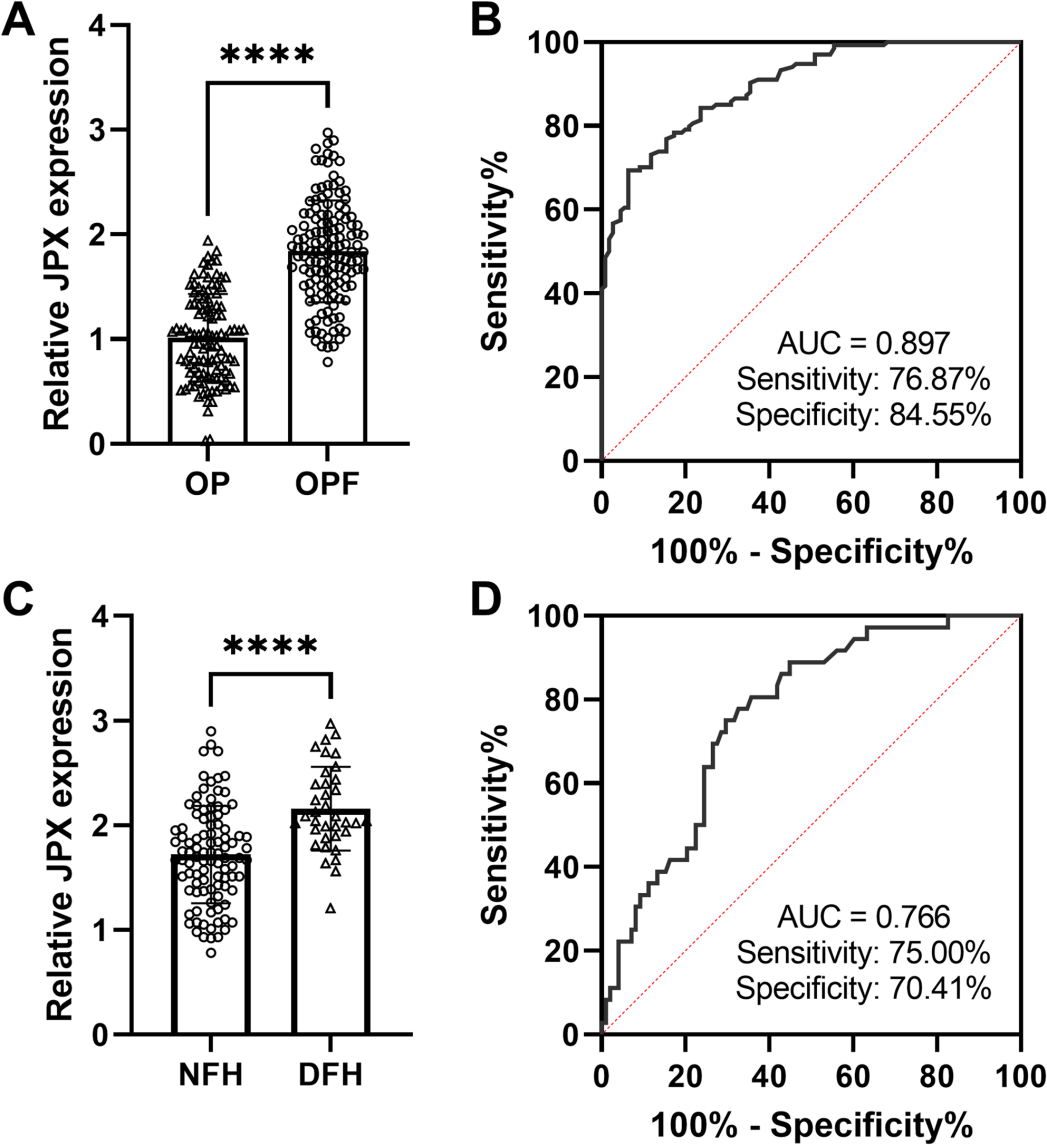
Expression of JPX in OPF and DFH and its diagnostic value. **A** JPX expression is increased in OPF. **B** ROC curve for JPX in OPF patients. **C** JPX expression is increased in DFH. **D** ROC curve for JPX in DFH patients. **** *P* < 0.0001

RT-qPCR showed that JPX expression was higher in OPF patients than in OP patients (*P* < 0.001, Fig. 1A). JPX demonstrated good diagnostic performance with an AUC of 0.897, specificity of 76.87%, and sensitivity of 84.55% (Fig. 1B). Multivariate logistic analysis identified JPX (95% CI 3.089–17.109; *P* < 0.001), T-score (95% CI 1.317–7.206; *P* = 0.009), and 25-(OH) vitamin D (95% CI 0.012–0.070; *P* < 0.001) as independent factors associated with OPF occurrence (Table 3). Moreover, JPX was higher in DFH patients compared with NFH patients (*P* < 0.001, Fig. 1C), and JPX also showed good diagnostic value for DFH with an AUC of 0.766, specificity 75.00%, and sensitivity 70.41% (Fig. 1D). Additionally, JPX was an independent factor for delayed healing in DFH patients (95% CI 3.188–25.517; *P* < 0.001, Table 4).

**Table 3:** Logistic regression analysis of risk factors for OPF

Indicators	OR	95% CI	*P*
Age	1.664	0.714–3.878	0.238
BMI	1.424	0.625–3.427	0.400
Gender	0.726	0.212–2.346	0.570
Smoking	0.594	0.184–1.915	0.383
Drinking	0.543	0.186–1.589	0.265
Family history of osteoporosis	3.125	0.804–12.145	0.100
Bone density (T score)	3.081	1.317–7.206	0.009
25-(OH) vitamin D	0.029	0.012–0.070	< 0.001
LncRNA JPX	7.270	3.089–17.109	< 0.001

**Table 4:** Logistic regression analysis of risk factors for DFH

Indicators	OR	95% CI	*P*
Age	1.961	0.778–4.944	0.154
BMI	1.456	0.593–3.575	0.412
Gender	0.914	0.304–2.744	0.870
Smoking	2.084	0.624–6.965	0.233
Drinking	0.514	0.150–1.766	0.290
Family history of osteoporosis	1.197	0.463–3.094	0.711
Bone density (T score)	3.249	1.213–8.701	0.019
25-(OH) vitamin D	0.493	0.191–1.273	0.144
LncRNA JPX	9.019	3.188–25.517	< 0.001

### JPX expression during osteogenic differentiation of hBMSCs

**Fig. 2:**
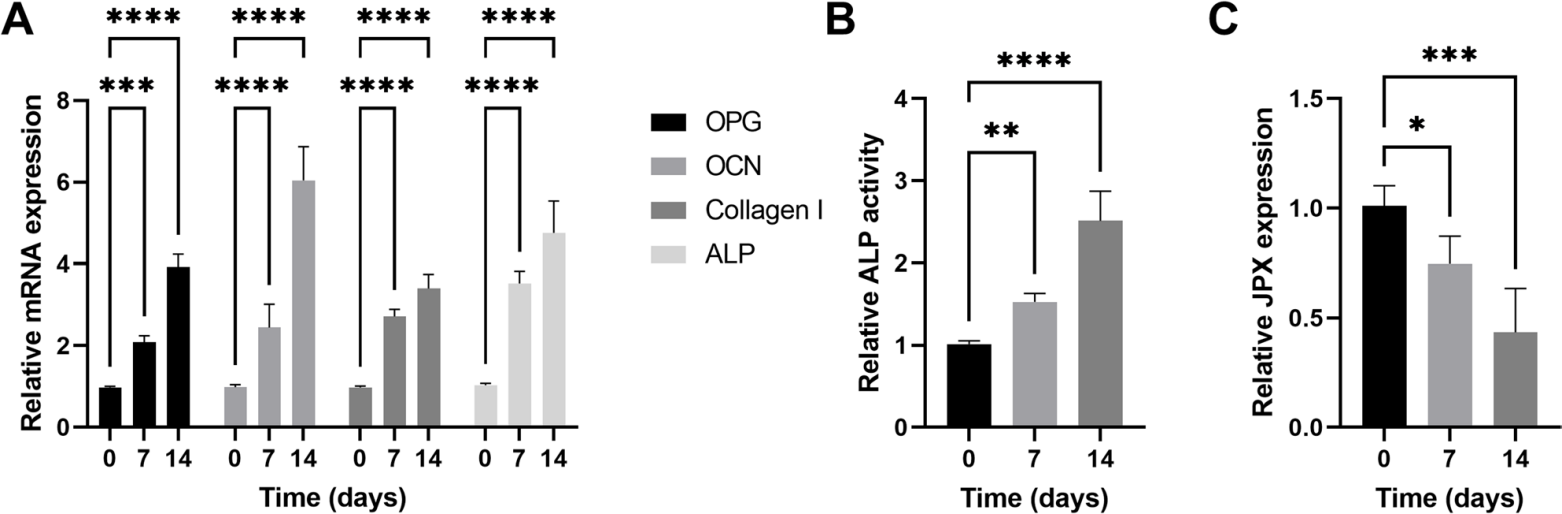
JPX expression during osteogenic differentiation of hBMSCs. As osteogenic differentiation progresses, the mRNA levels of osteogenic markers (OPG, ALP, Collagen I, and OCN) (**A**), ALP activity (**B**), and JPX levels (**C**) change. * *P* < 0.05, ** *P* < 0.01, *** *P* < 0.001, **** *P* < 0.0001

Osteogenic differentiation markers OPG, ALP, Collagen I, and OCN mRNA levels increased significantly as differentiation progressed (*P* < 0.001, Fig. 2A), and ALP activity showed a consistent significant upward trend (*P* < 0.01, Fig. 2B). The time points selected for this study (days 0, 7, and 14) were based on established protocols in the field [19, 20]. However, it should be noted that these sampling intervals may not have fully captured the true temporal profiles of the various biomarkers—for example, collagen I is typically rapidly upregulated during the early stages of differentiation. In contrast, JPX expression decreased significantly with progression of osteogenic differentiation (*P* < 0.05, Fig. 2C), suggesting a potentially key regulatory role for JPX in the pathological context of fracture delayed healing.

### Effects of JPX on osteogenic differentiation of hBMSCs

**Fig. 3:**
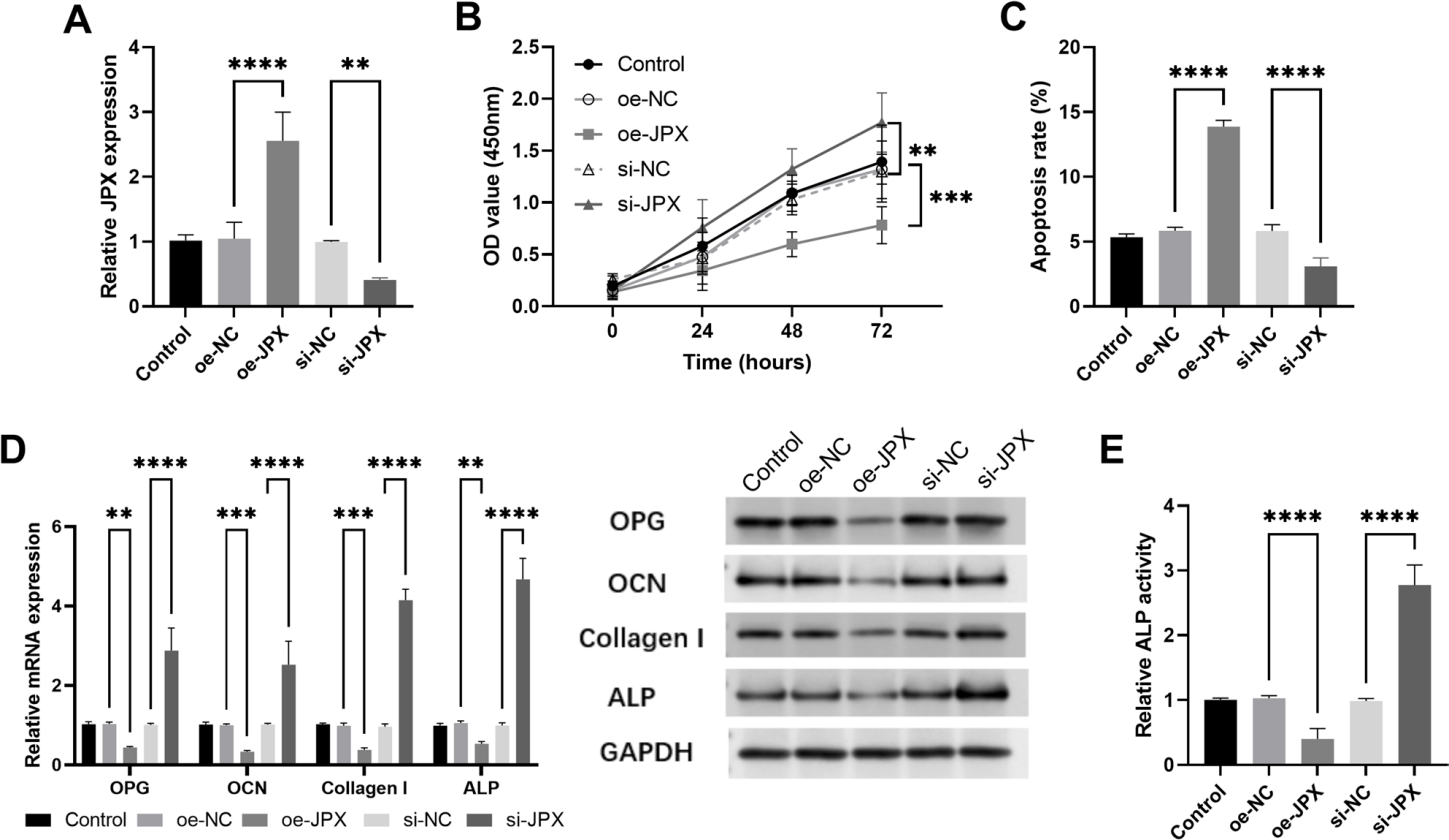
Effect of JPX on osteogenic differentiation of bone marrow mesenchymal stem cells (hBMSCs). Effects of transfection with oe-JPX and si-JPX on JPX levels (**A**), cell proliferation (**B**), apoptosis (**C**), osteogenic markers (**D**), and ALP activity (**E**). ** *P* < 0.01, *** *P* < 0.001, **** *P* < 0.0001

Overexpression of JPX (oe-JPX) and knockdown of JPX (si-JPX) were used to modulate JPX levels in hBMSCs (*P* < 0.01, Fig. 3A). Functionally, JPX upregulation reduced cell proliferation and increased apoptosis, whereas JPX inhibition restored proliferation and reduced apoptosis toward normal levels (*P* < 0.01, Fig. 3B and C). Assays related to osteogenic differentiation revealed that JPX overexpression significantly reduced the mRNA levels of OPG, ALP, Collagen I, and OCN, while ALP activity also decreased markedly; at the protein level, JPX overexpression similarly suppressed the expression of osteogenic markers (Collagen I , OCN, OPG, and ALP). Conversely, following JPX knockdown, both mRNA and protein expression of the aforementioned osteogenesis-related genes were significantly increased, and ALP activity was also enhanced (*P* < 0.01, Fig. 3D and E).

### JPX acts as a sponge for miR-219a-5p

**Fig. 4:**
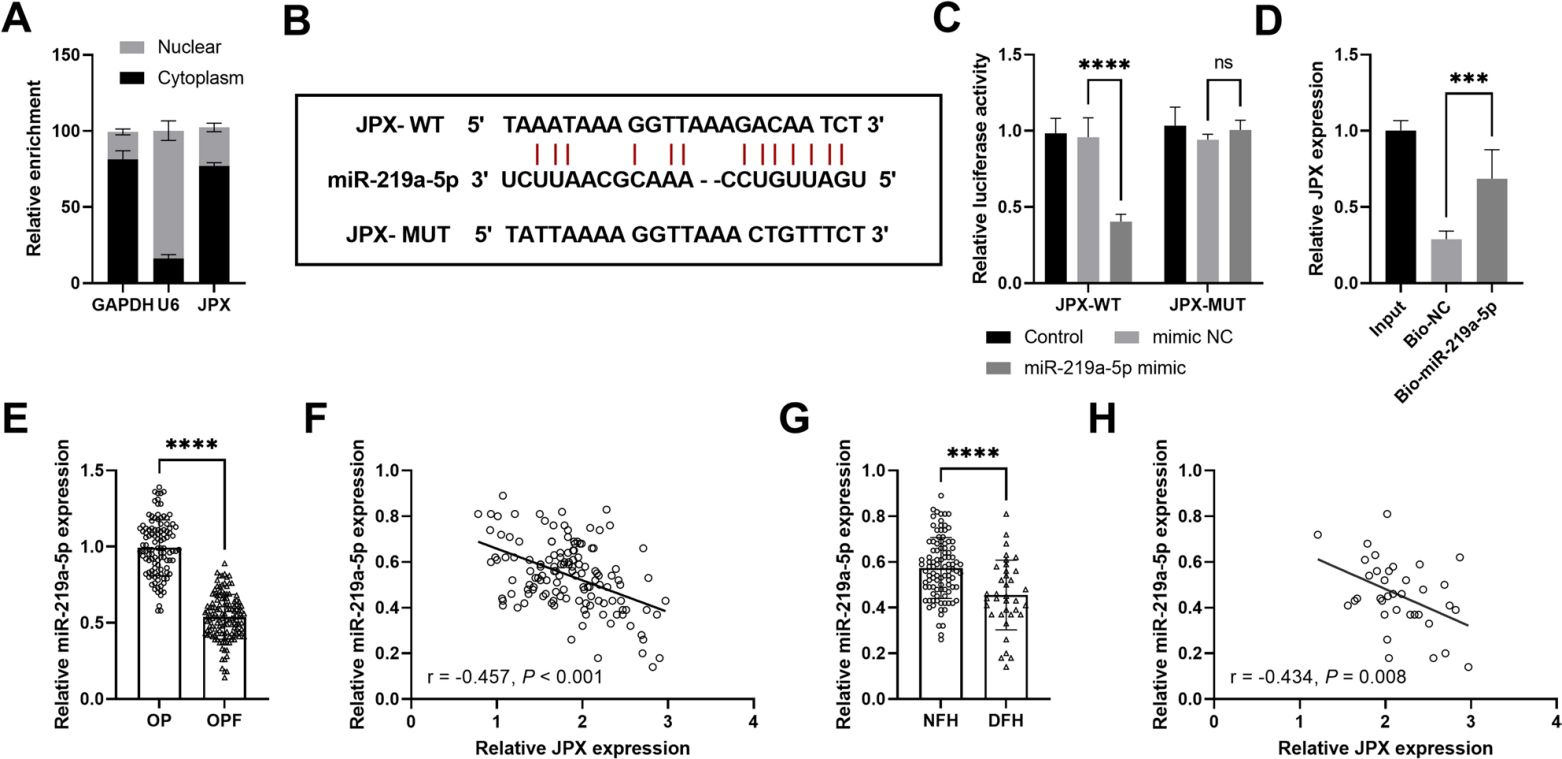
Sponge-like JPX–miR-219a-5p interactions. **A** Subcellular localization analysis of JPX. **B** Binding sites between JPX and miR-219a-5p. **C** Luciferase reporter assay. **D** RNA pull-down assay. **E** miR-219a-5p is decreased in OPF. **F** JPX and miR-219a-5p are negatively correlated in OPF. **G** miR-219a-5p is decreased in DFH. **H** JPX and miR-219a-5p are negatively correlated in DFH. *** P < 0.001, **** P < 0.0001

Subcellular localization analysis showed JPX predominantly localizes in the cytoplasm (Fig. 4A), suggesting potential as a competing endogenous RNA (ceRNA). Target prediction using ENCORI indicated a binding site between JPX and miR-219a-5p (Fig. 4B). Dual-luciferase reporter assays and RNA pull-down experiments further confirmed a direct interaction between JPX and miR-219a-5p (Fig. 4C and D). In clinical samples, miR-219a-5p expression was significantly lower in the OPF group than in the OP group (*P* < 0.001, Fig. 4E), and JPX and miR-219a-5p expression showed a significant negative correlation in OPF patients (*r* = − 0.457, *P* < 0.001, Fig. 4F). Moreover, miR-219a-5p expression was significantly lower in the DFH group than in the NFH group (*P* < 0.001, Fig. 4G), and their expression was also negatively correlated in DFH patients (*r* = − 0.434, *P* = 0.008, Fig. 4H).

### miR-219a-5p upregulation reverses the effects of JPX on hBMSCs

**Fig. 5:**
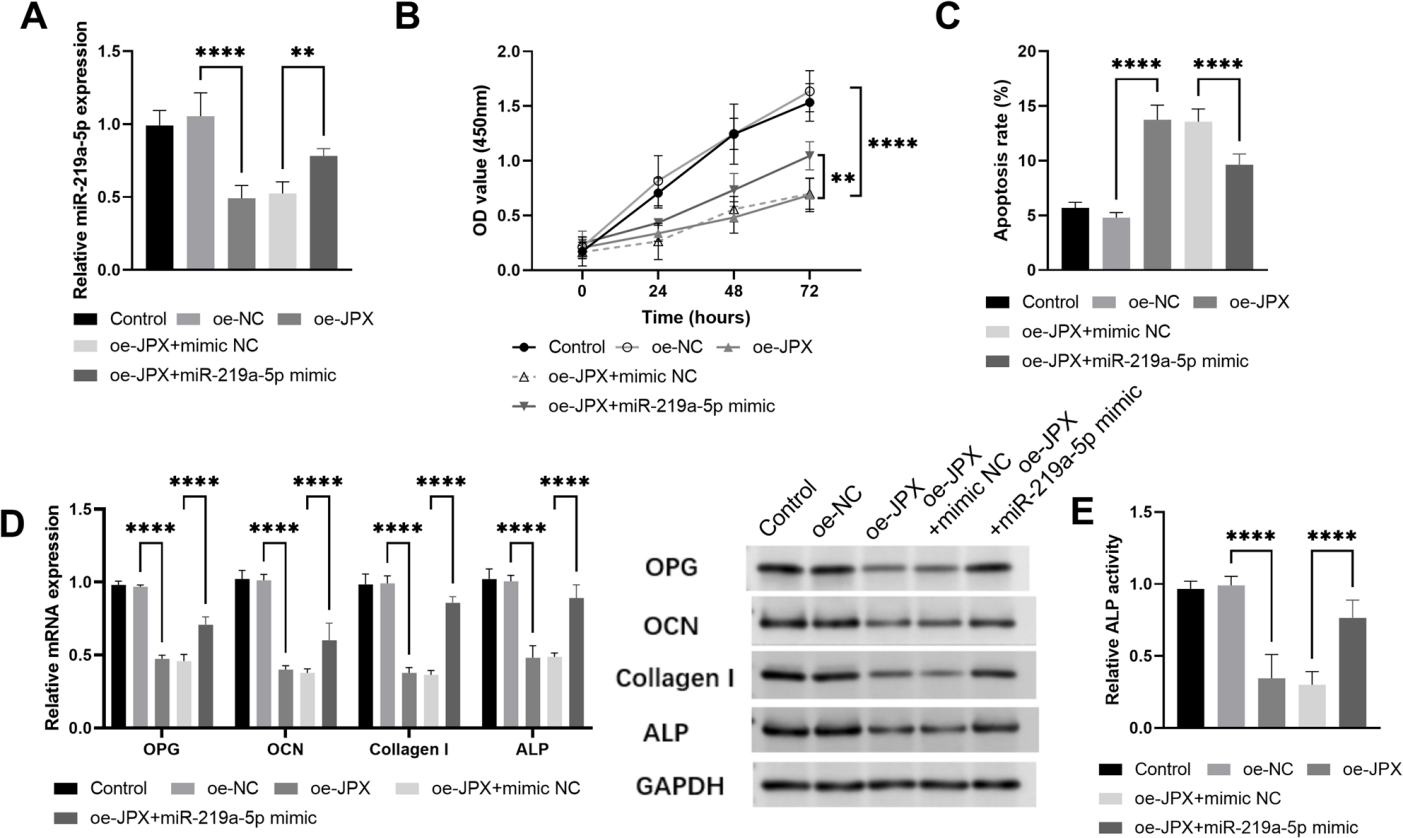
Upregulation of miR-219a-5p reverses the effects of JPX on hBMSCs function. Transfection with miR-219a-5p mimic antagonizes JPX’s effects on miR-219a-5p levels (**A**), cell proliferation (**B**), apoptosis (**C**), osteogenic markers (**D**), and ALP activity (**E**). ** *P* < 0.01, **** *P* < 0.0001

To further investigate the role of the JPX/miR-219a-5p axis in fracture healing, we performed co-transfection of miR-219a-5p mimic in the context of JPX overexpression. The results showed that JPX overexpression lowered the expression of miR-219a-5p, and co-transfection with the miR-219a-5p mimic significantly reversed this suppressive effect (*P* < 0.01, Fig. 5A). Furthermore, overexpression of miR-219a-5p antagonized JPX’s suppression of proliferation and its promotion of apoptosis (*P* < 0.01, Fig. 5B and C). More importantly, overexpression of miR-219a-5p significantly attenuated the inhibitory effect of JPX on osteogenic differentiation, as evidenced by a partial recovery in both mRNA and protein levels of osteogenic markers, accompanied by a marked increase in ALP activity. (*P* < 0.001, Fig. 5D and E).

## Discussion

OP patients face a higher risk of fractures, especially among postmenopausal women and men aged 50 and older [21]. OP leads to a decline in bone strength, making OPF possible even with minor traumas or daily activities, causing substantial inconvenience for patients [22, 23]. Moreover, the healing rate of OPF affects normal activity and psychological well-being [24]. Therefore, in-depth study of how lncRNAs regulate OPF and fracture healing is of great significance. This study investigates the molecular mechanisms underlying OPF and DFH through integrated analyses of clinical samples and in vitro models, first revealing the role and potential clinical value of the lncRNA JPX and miR-219a-5p axis in fracture healing.

The premise that lncRNAs exhibit tissue specificity, disease specificity, time specificity, and high stability supports their potential as ideal clinical diagnostic biomarkers [25]. For example, Ma et al. proposed PINT as an early diagnostic biomarker for DFH [26]. JPX is a 1696 bp long lncRNA transcribed from the X-inactivation center [27]. Multiple studies have reported aberrant JPX expression in various cancers [28, 29]. Our study first found that JPX is highly expressed in the serum of patients with OPF and DFH. Further analyses show that JPX demonstrates good diagnostic performance for OPF and DFH, with AUCs of 0.897 and 0.766, respectively. These results indicate JPX has high sensitivity and specificity, highlighting its potential as a diagnostic biomarker for OPF and DFH with broad clinical applications.

During fracture healing, as the process progresses, the number of osteoblasts increases, and their degree of differentiation and biological activity are markedly enhanced, thereby accelerating the pace of new bone formation [30]. Successful bone healing requires the highly coordinated interaction of multiple cell types and signaling pathways; any disruption of this coordination can lead to delayed healing or nonunion [31]. lncRNAs broadly participate in regulating cellular life activities [32], and thus may exert important regulatory effects on osteoblast function. Our study shows that JPX is upregulated in patients with OPF and DFH and is associated with poor fracture healing, suggesting that JPX may contribute to distorted BMSC behavior, impaired osteogenic function, and increased fragility. It is well known that increased ALP activity is a marker of osteoblast functional activation, driving bone formation by promoting the mineralization process [33]. Expression levels of other osteogenic markers (such as OCN, Collagen I, and OPG) and changes in cell viability can also serve as important indicators reflecting osteoblast activity and new bone formation [34]. We found that JPX upregulation functionally inhibits the proliferation and promotes the apoptosis of hBMSCs, and markedly suppresses osteogenic differentiation; conversely, JPX inhibition can protect osteogenesis. These results suggest that JPX is a negative regulator of osteogenesis. Mechanistically, JPX is primarily localized in the cytoplasm and supports ceRNA interactions, exerting its effects through regulation of miRNAs [35]. Numerous examples exist of lncRNA–miRNA interactions during osteogenesis; for instance, MEG3 can positively regulate osteogenic differentiation of hBMSCs by targeting miR-21-5p, thereby enhancing bone regeneration [36]. This study found that JPX directly interacts with miR-219a-5p, as indicated by bioinformatic predictions, luciferase reporter assays, and RNA pull-down experiments. Furthermore, a miR-219a-5p mimic can reverse the effects of JPX, indicating that miR-219a-5p mediates the osteogenic suppression and pro-apoptotic environment driven by JPX overexpression. These findings are consistent with the concept that lncRNAs shape bone fate through miRNA networks [37].

It must be acknowledged that this study has certain limitations. First, we observed the expression patterns of OPG, OCN, type I collagen, and ALP over a period of 14 days. Although the expression levels of these genes fluctuated, it is uncertain whether they have reached their peak expression. Due to the limited experimental period of 14 days and the lack of data at subsequent time points, we cannot confirm whether their expression levels will continue to rise, stabilize, or start to decline; therefore, it is possible that the maximum expression level was not captured within this time frame. Second, although the clinical sample size reached 244 cases, this was a single-center study, and the sample size for the DFH subgroup was relatively small; therefore, multicenter studies are needed to further validate the diagnostic value of JPX. Third, in vitro hBMSC models cannot fully simulate the complex in vivo microenvironment during fracture healing, and there is a lack of animal models for in vivo validation. Fourth, although this study confirmed the ceRNA mechanism of JPX/miR-219a-5p, the specific functions of miR-219a-5p downstream target genes in osteogenic differentiation still require further investigation. Future research will focus on the following directions: conducting longer-term observational studies to determine the true peak expression levels of these genes; conducting multicenter clinical studies to validate the diagnostic value of JPX; establishing animal models of fracture to validate the function of the JPX/miR-219a-5p axis in vivo; conducting in-depth screening and validation of miR-219a-5p downstream target genes; and exploring the potential of applications targeting this axis (e.g., via exosome delivery) to promote fracture healing.

In summary, this study demonstrates that JPX is upregulated in OPF/DFH and exhibits good diagnostic value. Functionally, JPX inhibits osteogenic differentiation and promotes apoptosis, thereby hindering fracture healing. Mechanistically, JPX acts through a ceRNA mechanism to sequester miR-219a-5p and disrupt the osteogenic microenvironment. Collectively, these findings point to the JPX/miR-219a-5p axis as a key regulatory node in fracture healing, offering potential diagnostic biomarkers and therapeutic targets.

## Supplementary Information

Supplementary Material 1.

## Data Availability

The datasets used and/or analyzed during the current study are available from the corresponding author on reasonable request.
