# Supplementary material for: Diagnostic value of LncRNA JPX in osteoporotic fracture (OPF) and its role in inhibiting osteogenic differentiation by targeting miR-219a-5p
Source: Hereditas. 2026 May 1;163:75. doi: 10.1186/s41065-026-00685-8 (PMC13289444; doi:10.1186/s41065-026-00685-8)
Supplement: Supplementary file 1 — Supplementary Material 1. [file 41065_2026_685_MOESM1_ESM.docx]

**Supplementary materials**

OPG


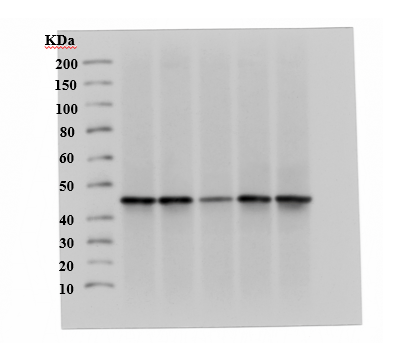


OCN





Collagen I





ALP





GAPDH





**Figure S1** The original image of Western blot of Figure 3D before clipping.



OPG



OCN

Collagen I





ALP





GAPDH





**Figure S2** The original image of Western blot of Figure 5D before clipping.
